# Stochastic Switching Induced Adaptation in a Starved *Escherichia coli* Population

**DOI:** 10.1371/journal.pone.0023953

**Published:** 2011-09-13

**Authors:** Yoshihiro Shimizu, Saburo Tsuru, Yoichiro Ito, Bei-Wen Ying, Tetsuya Yomo

**Affiliations:** 1 Graduate School of Information Science and Technology, Osaka University, Suita, Osaka, Japan; 2 Graduate School of Frontier Biosciences, Osaka University, Suita, Osaka, Japan; 3 Exploratory Research for Advanced Technology (ERATO), Japan Science and Technology Agency (JST), Suita, Osaka, Japan; University of Edinburgh, United Kingdom

## Abstract

Population adaptation can be determined by stochastic switching in living cells. To examine how stochastic switching contributes to the fate decision for a population under severe stress, we constructed an *Escherichia coli* strain crucially dependent on the expression of a rewired gene. The gene essential for tryptophan biosynthesis, *trpC*, was removed from the native regulatory unit, the *Trp* operon, and placed under the extraneous control of the lactose utilisation network. Bistability of the network provided the cells two discrete phenotypes: the induced and suppressed level of *trpC*. The two phenotypes permitted the cells to grow or not, respectively, under conditions of tryptophan depletion. We found that stochastic switching between the two states allowed the initially suppressed cells to form a new population with induced *trpC* in response to tryptophan starvation. However, the frequency of the transition from suppressed to induced state dropped off dramatically in the starved population, in comparison to that in the nourished population. This reduced switching rate was compensated by increasing the initial population size, which probably provided the cell population more chances to wait for the rarely appearing fit cells from the unfit cells. Taken together, adaptation of a starved bacterial population because of stochasticity in the gene rewired from the ancient regulon was experimentally confirmed, and the nutritional status and the population size played a great role in stochastic adaptation.

## Introduction

Living cells cope with stressful environments and adapt to changes in their surroundings using well-organised molecular machinery, such as signal transduction systems, operons, and regulons [1], [2], [3]. On the other hand, stochasticity in gene expression disturbs the precise control of gene expression [4], [5], and such noisy gene expression has been suggested to play a role in differentiation and adaptation of living organisms, alongside the regulatory mechanisms [6]. That is, the gene expression turns out to be randomly switching ON and OFF and is independent on any endogenous regulation. When the induced or suppressed expression happens to an essentially required gene, stochastic switching between ON and OFF states will have a great impact in fitness (*i.e.*, cell growth) and finally determine the cell fate. Such stochastic switching-induced adaptation, named as stochastic adaptation, has been proposed, but little supporting experimental evidence has yet been reported, in contrast to the large numbers of studies regarding the evolved molecular machinery-mediated adaptive responses.

Furthermore, pressure is generally exerted against the regulatory systems by genetic and environmental perturbations, which could alter the architecture of regulatory pathways and induce topological changes globally [7], [8]. Similarly, stochastic gene expression is also influenced by environmental stresses and genetic mutations [9], and the magnitude of noise in gene expression is quite different depending on gene categories or properties, *i.e.*, whether the gene is essential or nonessential [9], [10], [11]. It is unclear whether and how often population adaptation can be achieved once a gene essential for growth shows large fluctuations in expression but lacks a regulatory pathway.

To provide experimental confirmation of quantitative observations regarding stochastic switching stringently related to population survival, two requirements are essential: a gene crucial for adaptation in a stochastic manner and a switch-like genetic structure producing the fit and unfit phenotypes. As the known adaptive responses are usually under specific regulation [1], [2], [3], the stochastic mechanism for adaptation must generally be initially constructed in the lab. This is generally achieved by rewiring the gene from its native regulatory machinery to place it under extraneous control [12], [13], [14]. Only cells that can express the rewired gene are able to survive, because the adequate expression level of this gene is closely correlated to cell growth. This shuffling approach breaks the evolved regulatory mechanisms and provides a simple system in which to examine whether and how fluctuations in gene expression contribute to population adaptation.

With regard to the genetic structure, positive feedback gene networks are widely used [15], [16], [17]. The features of positive feedback circuits, monostable or bistable, can be easily modulated by chemical inducers, as the circuit generally involves promoter-repressor-inducer regulation [16], [17]. Bistability allows the cells to possess two stable states and shows fixation effect on the occasionally appeared states, which will greatly benefit for the experimental observation [15], [16], [17]. The transition between the two states occurs stochastically due to the noisy gene expression and facilitates the response to external cues [16], [18]. The frequency of so-called stochastic switching contributes greatly to the determination of cell fate and adaptation of the cell population [16], [17], [18], [19], [20], [21]. In the present study, we employed lactose utilisation network, as it is a well-characterised and commonly used as a native positive feedback circuit [16], [22].

We placed an endogenous gene, *trpC*, essential for tryptophan biosynthesis, into the lactose utilisation network in *Escherichia coli*. As *trpC* is one of the structural genes of the ancient regulatory machinery, the *Trp* operon [23], the lack of *trpC* expression generally disturbs cell growth in the absence of tryptophan [24], [25]. Rewiring of *trpC* from its native regulon (P_trpLp_ and P_trpCp_) [23], [26] and placing it under extraneous control (P_lac_) provided a model for stochastic switching between two gene expression states, one of which conferred a growth advantage under some growth conditions (-Trp) but not others (+Trp). In addition, employing the bistablility in the lactose utilisation network [16], [22], two stable phenotypes, *i.e.*, induced or suppressed expression of *trpC*, would be formed corresponding to tryptophan synthesis or not, respectively. As a consequence, cells in the induced state would survive tryptophan starvation, while those in the suppressed state would not.

According to theoretical expectations [16], [18], [19], [20], [21], the success in adaptation of the initially unfit cell population depends on two aspects, the switching rate of individual cells and the size of the unfit population. Thus, whether and how the switching rate changes in the unfit environment and the size of the population required for survival, were specifically addressed in our experiments. The results indicated that the rewired cells succeeded in growing under tryptophan-free conditions. However, the switching rate from the suppressed to induced state dropped off markedly due to tryptophan depletion, although the transition was supposed to be from the unfit to the fit state. The slow stochastic switching resulted in a risk of extinction, but was markedly compensated by the large population size. The efficiency of stochastic switching-mediated adaptation was dependent on not only the promoter activity but also on the nutritional status and population size.

## Results

### The rewired *trpC* implemented lactose utilisation network

**Figure 1:**
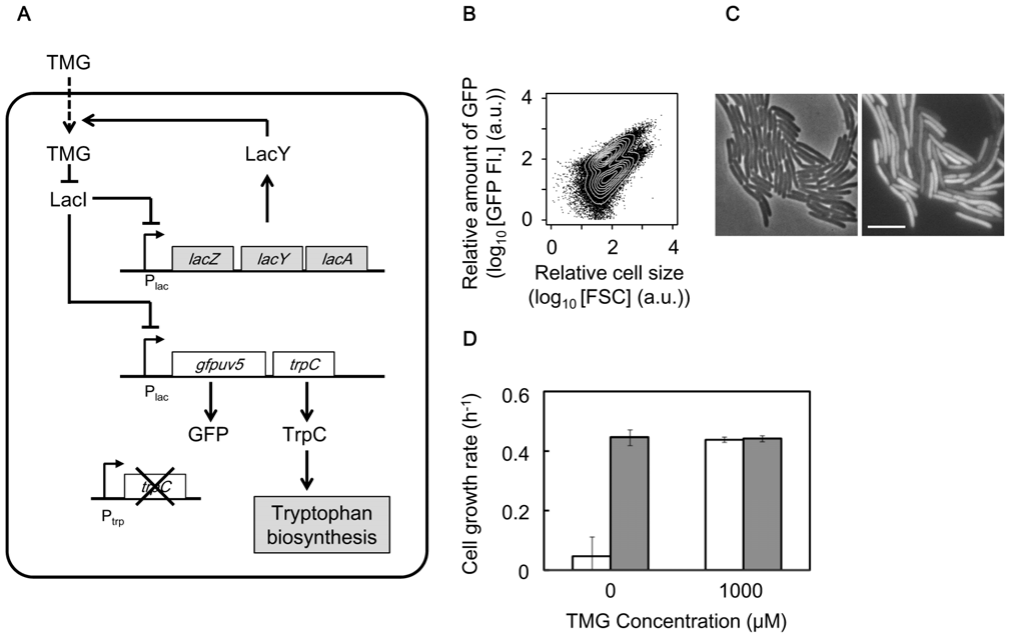
Rewired *E. coli* cell. **A**. Modified lactose utilisation network with the rewired *trpC*. Influx of TMG is mediated by lactose permease LacY. Lactose repressor LacI inhibits lactose promoter P_lac_ and is inactivated by TMG. *gfpuv5* and *trpC* coding green fluorescent protein and phosphoribosylanthranilate isomerase, respectively, under the control of P_lac_. **B**. Scatter plot merged with contours of the cell population in the presence of tryptophan with 300 µM TMG obtained by flow cytometry. GFP FI and FSC (forward scattering) represent the fluorescence of green emission from GFP and relative cell size, respectively. **C**. Microscopic images of cell populations indicated in **B**. The phase-contrast and fluorescent images are shown on the left and right, respectively. Scale bar, 5 µm. **D**. Growth rate of the induced or suppressed cells in the presence (closed bars) or absence (open bars) of tryptophan. The suppressed and induced cells were prepared in the absence of TMG and by induction with 1,000 µM TMG, respectively. Error bars represent standard deviation for 3 replicates.

The lactose utilisation network in *E. coli* is comprised of a three-node positive feedback loop (Figure 1A) consisting of the Lac repressor (LacI), the Lac permease (LacY) and the chemical inducer (thiomethyl-β-galactoside, TMG). The gene essential for tryptophan biosynthesis, *trpC*, was replaced from the tryptophan operon to another chromosomal location (the position of *intC*), and co-expressed with the reporter gene, *gfp*uv5, encoding the green fluorescence protein. The expression of *trpC* and *gfpuv5* was controlled by the Lac promoter (P_lac_), and was taken to be synchronised with the native lactose utilisation network due to the identical regulatory structure. In the presence of the inducer (*i.e.*, 300 µM TMG), two discrete subpopulations with induced and repressed expression of GFP (TrpC) were observed by flow cytometry (Figure 1B) and microscopy (Figure 1C, Figure S1). The expression of the rewired *trpC* was quantified by monitoring the level of GFP (green fluorescence). The genetic rewiring (*trpC* replacement) did not alter the generation time (growth rate) in the presence of tryptophan in comparison to the control strain that had *trpC* under its native regulation (data not shown). The cell growth rate was markedly reduced once tryptophan was depleted in the medium, but recovered in the presence of 1,000 µM TMG (Figure 1D). These observations verified that induction of the rewired *trpC* was able to compensate for the damaged *Trp* operon and rescue cell growth.

**Figure 2:**
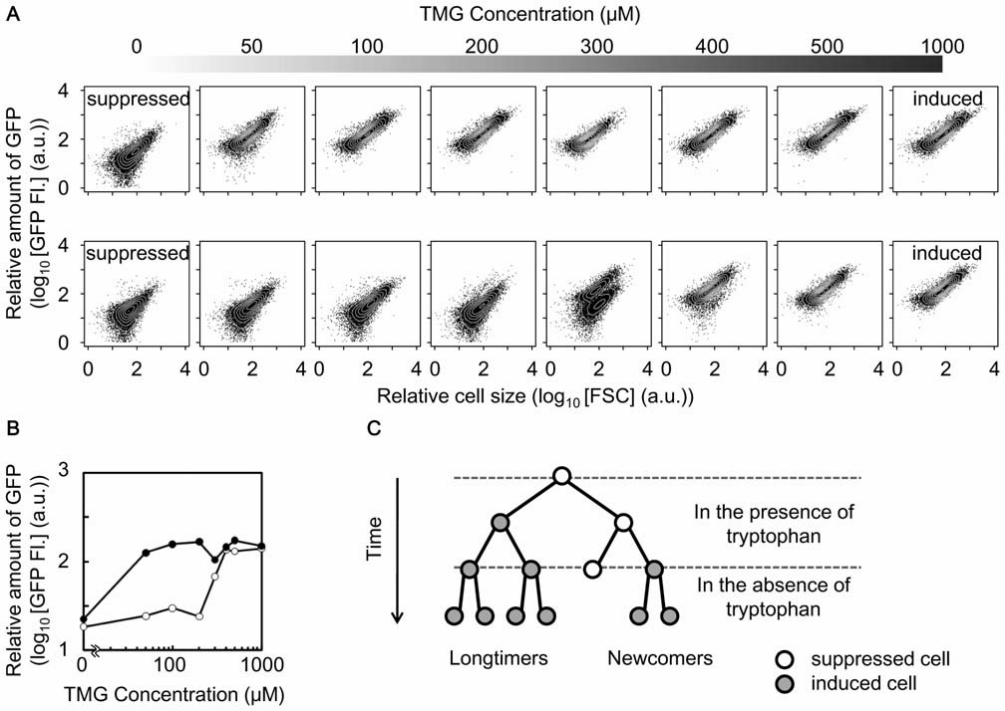
Hysteresis and bistability of the induced and suppressed states. **A**. Scatter plots merged with contours of cell population with various concentrations of TMG. The top grey bar represents the concentration of TMG as greyscale and each plot indicates the cell population exposed to the corresponding TMG concentrations 18 h after inoculation. The upper and lower panels indicate the cell populations preliminarily cultured in the presence (induced state) and absence (suppressed state) of TMG, respectively. The induction was carried out with 1,000 µM TMG. **B**. Hysteresis in the expression of GFP (TrpC). The relative concentrations of GFP (TrpC) were calculated as the mean values of the cell populations indicated in **A**. The black and grey trajectories represent the cell populations of the suppressed (upper panels in **A**) and induced (bottom panels in **A**) memories, respectively. **C**. Schematic diagram of cellular lineage before and after tryptophan depletion. The induced cells (grey circles) grew even when tryptophan was depleted, while the suppressed cells (open circles) did not proliferate unless the stochastic switching event occurred. Colour change in the cellular lineage diagram corresponds to the change in expressional change for a particular cell.

Hysteresis and bistability of the *trpC* implemented lactose network were examined subsequently under tryptophan-supplemented conditions. Cells preliminary grown in the presence (1,000 µM) or absence of the inducer (TMG) were transferred to fresh media containing varied amounts of TMG from 0 to 1,000 µM. Diverse distributions of GFP level dependent on the initial state (induced or suppressed) of the cells were clearly observed (Figure 2 A–B). The cells preliminarily grown under the induced conditions (Figure 2A, top panel) maintained the high expression level (*i.e.*, the induced state) regardless of whether the concentration of TMG was as low as 50 µM. In contrast, the cells without preliminary induction (Figure 2A, bottom panel) formed a population with a low expression level (*i.e.*, the suppressed state) up to 100 µM TMG. In addition, bimodal populations showing the coexistence of cells in the suppressed and induced states were observed with intermediate concentrations of TMG (200 – 500 µM). These results confirmed that the positive feedback structure of the modified gene network provided two stable states (*i.e.*, bistability) for the cells—the induced state with high expression of *trpC* and the suppressed state with repressed *trpC* expression.

In summary, the bistability fixed the cells at the induced or suppressed level of TrpC (GFP). The bimodal distribution of GFP (TrpC) level (Figure 1 B–C) caused by the noisy gene expression of the positive feedback circuit (Figure 1A) indicated the fit and unfit states once tryptophan was absent (Figure 1D, Figure 2C). We hypothesize that stochastic switching between the two states could stochastically activate the expression of *trpC* and may provide a mechanism for adaptation to tryptophan limitation in the rewired cells. Accordingly, we assumed that only the rewired cells switching to the induced state could survive starvation and the switching rate from the unfit to the fit state greatly influences the chance of survival (Figure 2C).

### Population transition in response to tryptophan depletion

**Figure 3:**
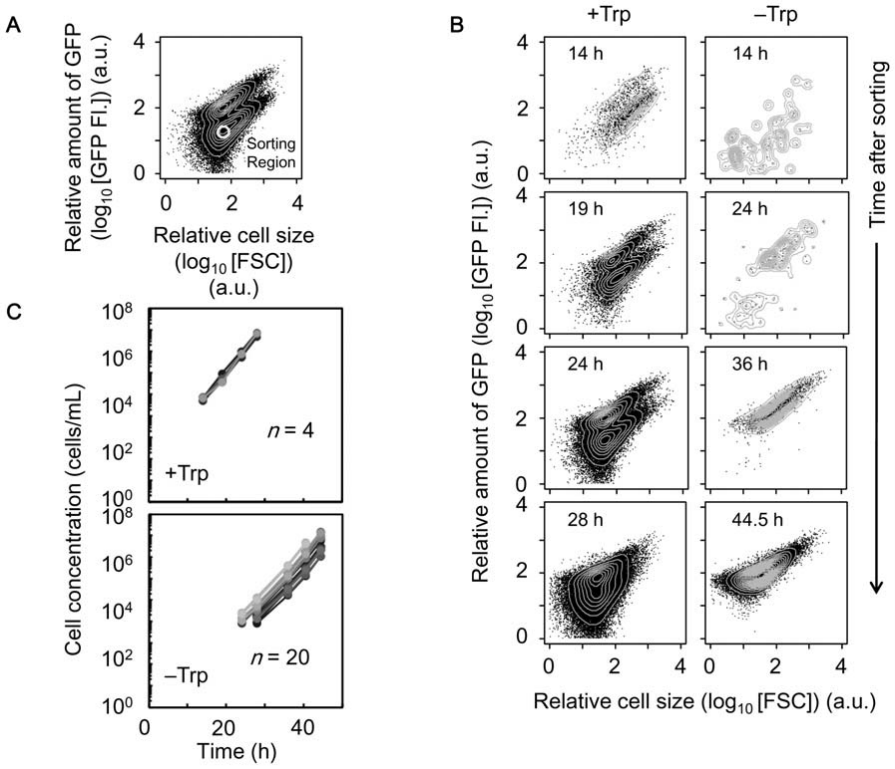
Population dynamics in the presence and absence of tryptophan. **A**. Sorting region (white circle) of suppressed cells in the presence of tryptophan with 300 µM TMG. One hundred suppressed cells were sorted into each nutrient condition in the presence or absence of tryptophan and further cultured. **B**. Temporal changes in the cellular states in the presence (+Trp) and absence of tryptophan (−Trp) with 300 µM TMG. The insets indicate culture time after sorting. **C**. Growth curves of cell populations in the presence (+Trp) and absence (−Trp) of tryptophan with 300 µM TMG after cell sorting. The differences in greyscale represent replicates (*n* = 4 and 20 in the presence and absence of tryptophan, respectively).

To address the question in our hypothesis, that is, whether the induced cells would be born from the suppressed population, whether the cells could survive from starvation, and how often such adaptation occurs (Figure 2C), stochastic switching-induced population transition was observed using a cell sorter. Samples of 1,000 cells with repressed expression were sorted from the bimodal populations exposed to 300 µM TMG in the presence of tryptophan (Figure 3A), and inoculated into fresh medium in the presence or absence of tryptophan with the same induction of 300 µM TMG (Figure 3B). Due to the stochasticity of switching, multi-well dishes were used to obtain a reliable switching rate (4 and 20 wells for estimation of switching rate in the presence and absence of tryptophan, respectively) (Figure 3C). Timed sampling of both cultures was performed to record the cell concentration and relative expression level (green fluorescence, GFP FI.). In the presence of tryptophan, the suppressed cells grew exponentially, along with the stochastic appearance of the induced cells (Figure 3 B–C, +Trp). The bimodal population, roughly restored as the initial population (Figure 3A), formed again 24 h later (Figure 3B, +Trp).

In contrast, the unimodal distribution of the induced population appeared in the absence of tryptophan (Figure 3B, −Trp). As the inoculated cells were selected from the repressed subpopulation (Figure 3A, sorting region), the induced cells must have been derived from the initial 1,000 suppressed cells and subsequently proliferated due to the high expression level of *trpC* (Figure 3C, −Trp). In addition, this population transition was clearly observed under other conditions (*e.g.*, 400 µM TMG) (Figure S2). The results showed that the switch from the suppressed to the induced state occurred and it permitted the rewired cells to adapt to the tryptophan-free conditions. The observations indicated that the cells could survive from starvation by means of stochastic switching, although the required gene was outside the regulation by the native operon.

### Reduced switching rate under tryptophan starvation

**Figure 4:**
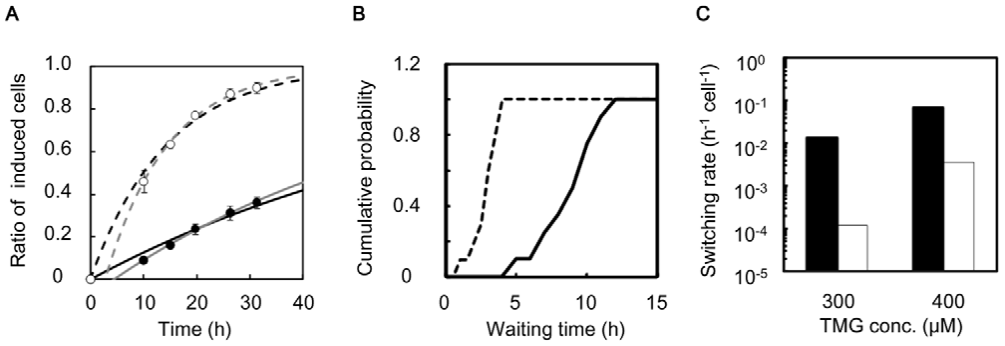
Switching rate in the presence and absence of tryptophan. **A**. Temporal change in the ratio of the number of induced to suppressed cells in the presence of tryptophan with 300 µM (filled circles) and 400 µM (open circles) TMG. The solid (300 µM) and broken (400 µM) lines are the fitting curves according to the population dynamics model (Materials and Methods). Gray lines represent the exponential fit when considering the lag time. The switching rates were calculated as 0.014 (solid line in black), 0.070 (broken line in black), 0.017 (solid line in gray) and 0.085 h^−1^cell^−1^ (broken line in gray), respectively. Error bars show the standard deviations. **B**. Cumulative distribution of the wait time of the first induced cell born from the initial population of 1,000 or 100 suppressed cells, in the absence of tryptophan with 300 (solid line) or 400 µM (broken line) TMG, respectively. The average wait times were 8.6 h for 300 µM TMG and 2.7 h for 400 µM TMG. **C**. Switching rates in the presence (filled bars) and absence (open bars) of tryptophan. The switching rate was determined as the inverse of wait time divided by the number of cells in the initial population; *e.g.*, 0.00012 h^−1^cell^−1^ = 1/8.6/1,000.

As the switching occurred stochastically, the frequency of the transition from the unfit to the fit state must be crucial for survival. The switching rate (h^−1^cell^−1^) was subsequently estimated according to the population dynamics model [19]. In the presence of tryptophan, the ratio of induced cells in the population was calculated by transforming the bimodal distribution (*e.g.*, Figure S3 A). Temporal changes of the ratio were captured accordingly (Figure 4A, Figure S3 B). Here, the time scale reflected the switching rate from the suppressed to induced state, as both cellular states showed the equivalent growth rate (*e.g.*, Figure 1D, Figure 3B). The switching rates were 0.070 and 0.014 h^−1^cell^−1^ under the inductions of 400 and 300 µM of TMG, respectively (increased to some extent once considering the slight delay, Figure 4A, gray lines). In the absence of tryptophan, the wait time for the first new induced cell reflected the switching rate. The variation in the exponential growth curves (Figure 3C, −Trp, among 20 wells) suggested the alternative stochasticity in the transition wait time. The wait time could be estimated from the horizontal intercept of the exponential growth of the induced cells. The cumulative distribution of the wait time was shown based on experimental data from 20 wells (Figure 4B). The average wait time was approximately 8.6 h with the initial pool of 1,000 suppressed cells, resulting in a switching rate of 0.00012 h^−1^cell^−1^ (details in Materials and Methods). These observations showed that the switching rate was reduced markedly when tryptophan was depleted (Figure 4C). In another word, the rate of transition from suppressed to induced cells was enhanced in growing cultures (+Trp) compared to non-growing cultures (−Trp). It seemed that the transition from the unfit to the fit state was not favoured by starvation when the cells were exposed to severe stress. Note that the starved cells kept being viable at least for 20 h (Figure S4), which was longer than the waiting time (∼8.6 h) for the emergence of the first induced cell.

### Large population size compensated for slow switching

To understand the influence of such a reduced switching rate on adaptation, we examined the relation between the initial population size and the probability of adaptation. Theoretically, a smaller population size would be associated with fewer survival chances as the appearance of the fit cells per population (h^−1^population^−1^) was dependent on stochastic switching rate (h^−1^cell^−1^) and the population size (cells/population). The suppressed cells, which were grown in the presence of tryptophan and exposed to various levels of TMG (from 200 to 500 µM, Figure 2A, bottom panel), were sorted into fresh media containing the same level of TMG but without tryptophan. One, 10, 100 and 1,000 cells were collected as the initial populations under each culture condition. As a control, the induced cells, exposed to 200 µM TMG with a history of 1,000 µM TMG pre-induction (Figure 2A, upper panel), were also sorted into fresh media containing the same amount of TMG but without tryptophan. Twelve replicates of each population size under each condition (3 for control) were monitored for 1 week. Growth in each well was identified by eyes and the number of the well of growing cells was counted (details in Materials and Methods). Note that longer incubation time did not alter the results.

**Figure 5:**
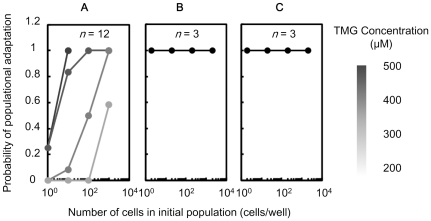
Persistence of population adaptation against reduced population size. Probability of adaptation of the cell population, ratio of wells with cell proliferation to total wells, is plotted over the number of cells in the initial population (population size) after collection through cell sorting. **A**. Probability of population adaptation for suppressed cells exposed to different TMG concentrations (from 200 to 500 µM) in the absence of tryptophan. The TMG concentration is shown in greyscale. **B**. Probability of population adaptation for induced cells, exposed to 200 µM TMG but with a history of 1,000 µM TMG in advance (Figure 2, upper panel), in the absence of tryptophan with the same TMG concentration. (c) Probability of populational adaptation for suppressed cells, exposed to 200 µM TMG, in the presence of tryptophan with the same TMG concentration.

The probability of population adaptation was evaluated as the ratio of the number of wells with growing cells to the total well number. The probability of population adaptation was positively correlated with both the population size and increasing TMG concentration (Figure 5A). For example, only half of the cells in the 100-cell populations showed propagation in the presence of 300 µM TMG, while all those in the 1,000-cell populations proliferated (Figure 5A, grey line). When the TMG concentration was raised to 400 µM, all cells in the 100-cell populations showed adaptation (Figure 5A, dark grey line). In contrast, all cells in the populations starting from induced cells proliferated independent of the population size (Figure 5B). Lack of dependence on population size was also observed when sorting the suppressed cells (Figure 2A, bottom panel) to tryptophan-containing medium in the absence of TMG (Figure 5C). As the switching rate was reduced by one hundredfold due to tryptophan depletion (Figure 4C), the relatively large population size was crucial for sustaining such slow switching populations and to wait for the appearance of fit cells. In summary, the quantitative relation between the population size and the survival chance not only validated the markedly reduced switching rate but also verified the impact of the population size to sustenance.

## Discussion

We modified the lactose utilisation network by introducing the rewired *trpC*, an essential gene for tryptophan biosynthesis, into a positive feedback circuit. The rewired cells formed two discrete phenotypes, the induced and suppressed states, with regard to respect to the fit (tryptophan synthesis) and unfit (no synthesis) states under tryptophan-depleted conditions, particularly in the presence of 300 µM TMG. Our study showed that stochastic switching from the suppressed to the induced state rescued the cell population under conditions of tryptophan starvation, and the switching rate was inconstant and highly dependent on external tryptophan. Both nutritional status and population size played crucial roles in the success of stochastic switching-mediated adaptation.

**Figure 6:**
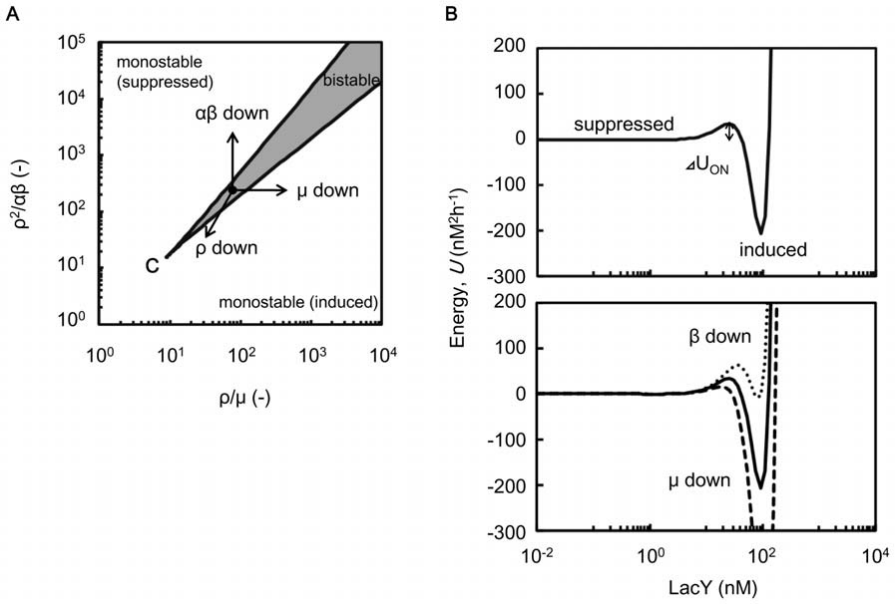
Parametric phase plane and energy landscape. **A**. Parametric phase plane of lactose utilisation network. Boundaries between monostable and bistable (grey) regime was calculated by the general positive feedback model (Materials and Methods). The critical point is indicated by C. Parameters *µ* and *α* are the cell growth rate and the maximal synthesis rate of LacY, which is achieved when every LacI repressor is inactivated, respectively. The other two parameters, *β* and *ρ*, are proportional to TMG uptake rate per LacY and synthesis rate of LacI, respectively (Materials and Methods). **B**. Example of energy landscape respective to LacY in the bistable regime. The energy landscape was calculated by integration of the differential equation of LacY with regard to LacY (solid lines, Materials and Methods). The energy barrier from the suppressed to the induced state is indicated by *ΔU_ON_*. The energy landscapes with reduced *β* and *µ* are also indicated in the bottom panel (dotted and dashed lines, respectively). The parameters are as follows: *α*, 60 nM h^−1^; *ρ*, 50 h^−1^; *β*, 0.1 (solid and dashed lines) or 0.09 (dotted lines) µM^−1^h^−1^; *µ*, 0.5 (solid and dotted lines) or 0.45 h^−1^ (dashed lines).

The regulatory network evolved for lactose utilisation, comprised of lactose repressor, Lac promoter, lactose permease and lactosidase, is generally activated under conditions of glucose depletion and/or lactose abundance [2]. As a consequence, switching between the two states is widely observed in response to glucose [16]. Here, we performed experiments under conditions of glucose starvation using the same rewired strain. A relatively high switching rate (0.083 h^−1^cell^−1^) was observed (Figure S5), in good agreement with the results reported previously [16]. These observations raise questions regarding why the switching rate decreased in response to tryptophan depletion. We refined the parameters involved in the modified lactose utilisation network. Amino acid starvation usually down-regulates the transcription/translation reaction, leading to a decline in growth [8], [27]. The parameters involved in these reactions apparently contributed to the network state. The parametric phase plane of lactose utilisation network (Figure 6A) described the relation between the biological parameters and the network states (*i.e.*, induced or suppressed, monostable or bistable). ρ, µ, α and β represent LacI production (transcription/translation) rate, cell growth (elongation) rate, maximal production rate of LacY (GFP, TrpC) and TMG uptake rate per LacY molecule, respectively (see Materials and Methods). As shown in the energy landscape (Figure 6B), the switching rate from the suppressed to the induced state relied on the height of the energy barrier. Accordingly, the reduced cell growth rate (µ) and rate of LacI production (ρ) potentially biased the network to the induced state with the reduction of the energy barrier from suppressed to induced, resulting in an increase in switching rate (Figure 6). On the other hand, reduced rates of LacY production (GFP, TrpC) (α) and of TMG uptake rate per LacY molecule (β) potentially biased the network to the suppressed state with the increase in the energy barrier, resulting in a decrease in switching rate (Figure 6). Thus, the switching rate was determined by the balance among these contradictory parameters.

The present study showed that the switching rate dropped off considerably despite the reduced growth rate due to tryptophan depletion (Figure 4), suggesting that the frequency of stochastic switching could be influenced by the cellular stress due to the external environments. Other related questions, such as, whether the stress (*e.g.*, starvation) would contribute to the magnitude of noise, are intriguing and required to be addressed in the future. In addition, the results presented here suggested that the lactose utilisation network was able to inhibit the activity of the Lac promoter once the amino acid had been depleted, which may have been the resulted of global regulation, such as the stringent response [8], [27]. We postulated that other promoters (or gene networks) relatively robust to the environmental changes may modulate the switching rate through the growth decline [22], [28]. That is, a decrease in growth rate would not always result in a reduction in switching rate. The characteristics of the genetic structure should be taken account. Thus, studies of other genetic structures and environments are essential to determine the general nature of the switching rate.

In the present study, we focused on the stochastic switching in the adaptive direction (from suppressed to induced). The reverse direction, *i.e.*, switching back from the induced to the suppressed state, is theoretically possible by modulating the network state. Recently, Acar *et al*. introduced two types of switching, *i.e.*, fast and slow switchers [19]. The fast switchers showed high switching rates in both directions, that is the frequent appearance of fit cells from the unfit population, accompanied by an increase in unfit cells newly derived from the fit cells. The rapid switch between the two stable states may be beneficial for the cell population exposed to frequently changing environments, avoiding the costly emergence of unfit cells. In contrast, the slow switchers showed slow switching rates in both directions, and thus either fit or unfit cells rarely emerged. The slow switching population may favour stable environments with low cost. Nevertheless, the slow switchers could avoid extinction with frequently perturbations by increasing the population size. As shown in Fig. 5, the smallest initial population size essential for survival varied according to the switching rate; the slower the switching rate, the larger the population size is required. A large population of slower switchers would successfully overcome external perturbations, regardless of whether they were either frequent or rare. In contrast, a limited number of cells would be sufficient for fast switchers to adapt to frequent environmental fluctuations. It indicated that diverse switching rates would be advantageous for the environments with different frequencies of perturbations and the limitation in population size to overcome non-preferential conditions. In summary, the present work showed that stochastic switching rate decreased significantly due to starvation, and demonstrated that large population size contributed greatly to survival chance for slow switchers. Stochastic switching mediated adaption was quantitatively evaluated here, and we believe it must be valuable for understanding of population sustenance in the wild nature.

## Materials and Methods

### Genetic construction of the bacterial strain

The *E. coli* strain DH1 was used for genome recombination. DH1*ΔtrpC* was constructed by homologous recombination, as described previously [25], [29]. The plasmid pLacG-*trpC* (a pBR322 derivative) containing the sequence of *P_lac_-gfp-trpC-P_kan_-kan* was constructed by inserting the gene *trpC*, which was amplified from the genome of *E. coli* DH1, into pGCND5T7T1T2_KN, at the *Hin*dIII site. The plasmid pGCND5T7T1T2_KN was constructed by introducing the fragments of rrnBT1T2 and *P_kan_-kan* into pGCND5T7 [30]. The terminator sequence rrnBT1T2 was amplified from pTrc99A (Amersham Pharmacia) and inserted into the *Pvu*II site (upstream of *P_lac_*). The sequence *P_kan_-kan* with *FRT*-flanked sites was amplified from pKD13 [29] and inserted into the *Not*I site. The PCR product of the target sequence *P_lac_-gfp-trpC-P_kan_-kan* amplified from pLacG-*trpC* with the primers intC-in-nt-r1 (5′-GCACTGGATTGCAAGACTTTGTGCTATTCGATAGTTGTTAAGGTCGCTCACTCGGCACGA-3′) and intC-in-Fn (5′-CCGCAAAATCCCCTGAATATCAAGCATTCCGTAGATTTACAGTTCGTCATGGGGATATAG-3′) was integrated into DH1*ΔtrpC* at the chromosomal location of *intC*, as described elsewhere [30]. The final rewired strain was designated as DH1*ΔtrpCΔintC*::*P_lac_-gfp-trpC-P_kan_-kan*.

### Cell culture

Bacterial cells were grown in minimal medium (modified M63, mM63: 62 mM K_2_HPO_4_, 39 mM KH_2_PO_4_, 15 mM (NH_4_)_2_SO_4_, 2 µM FeSO_4_•7H_2_O, 15 µM thiamine hydrochloride, 203 µM MgSO_4_•7H_2_O and 22 mM glucose) [25] in the presence or absence of 0.5 mM tryptophan. Cells were cultured at 37°C for several passages until the growth rate became stable. Exponentially growing cells were subsequently transferred to fresh medium supplemented with various concentrations of thiomethyl-β-galactoside (TMG), to induce the expression of LacY, GFP and TrpC. We used 300 and 400 µM TMG for determination of switching rate and evaluation of persistence to reducing population size as detailed below. For evaluation of hysteresis, bacterial cells initially cultured with 0 and 1,000 µM TMG were inoculated into fresh media with various concentrations of TMG (0, 50 100 and 1,000 µM). Cell cultures were examined by flow cytometry after 18 h (∼10^7^ cells/mL). The initial cell concentration was approximately 10^2^ cells/mL except for inoculation through cell sorting.

### Microscopic observations

Bacterial cells in the logarithmic growth phase (2 µL of culture) were placed on glass coverslips and immersed in the same medium as used for preculture. The cells were subsequently covered with a thin agarose pad (1.5%). Fluorescence images were acquired at 375× magnification using a fluorescence microscope (TE2000; Nikon) and a cooled CCD camera (DV887; Andor). The gain value was 1,000 and exposure time was 800 ms. Fluorescence from GFP was collected through a 500 – 540 nm emission filter.

### Flow cytometry

GFP expression (fluorescence intensity) and relative cell size were evaluated using a flow cytometer (FACSAria cell sorter; Becton Dickinson) with a 488 nm argon laser and a 515 – 545 nm emission filter (GFP). The following PMT voltage settings were applied: forward scatter (FSC), 203; side scatter (SSC), 440; GFP, 1,000. The flow data were analysed by custom-designed scripts written in R [31]. Systematic errors resulting from events that occurred at the bottom or top of the instrument's range were eliminated. Cell samples mixed with fluorescent beads (3 µm Fluoresbrite YG Microspheres; Polysciences) were loaded for calculation of cell concentration. For cell sorting, the bimodally distributed cells were sorted into the suppressed cells according to their green fluorescence intensity. The sorting gate was set to a narrow region around the median fluorescence of the suppressed population.

### Tryptophan depletion

Bacterial cells were grown in mM63 medium supplemented with 0.5 mM tryptophan and 300 µM TMG for 24 h as described above. Aliquots of 300 µL of exponentially growing cells (about 10^6^ cells/mL) were harvested by centrifugation at 5000 rpm (or 2300 × *g*) for 30 s at 37°C using spin columns (0.2 µm Ultrafree-MC Centrifugal Filter Units; Millipore). After discarding the flow-through fraction, the cells were washed with 300 µL of the same medium minus tryptophan. After repeating centrifugation and washing processes twice, the concentration of the cell suspension was determined by flow cytometry and sorted into 1 mL of the same medium with or without tryptophan. The initial cell concentration was 10^3^ cells/mL for measurement of switching rate, and 1 to 10^3^ cells/mL for evaluation of population size-dependent adaptation.

### Measurement of switching rate

Bacterial cells were sampled and analysed by flow cytometry over time after sorting into mM63 supplemented with 300 µM TMG with or without 1 mM tryptophan, where sorted cells were collected in multi-well dishes. To improve reliability, 4 and 20 populations were analysed for the presence and absence of tryptophan, respectively, where a large number of populations was analysed for the absence of tryptophan as estimation of the switching rate strongly depended on the stochastic emergence of the first induced cell in the population. The numbers of induced and suppressed cells in the population were calculated by fitting with GFP distribution of induced cells exposed to 1,000 µM TMG and that of suppressed cells without TMG (Figure S3). To avoid over- or underestimation associated with the commingling from suppressed (or induced) to induced (or suppressed) cells, which is mainly due to the positive correlation between cell size and GFP fluorescence, we transformed GFP distributions appropriately as detailed in Figure S3. To estimate the switching rate in the presence of tryptophan, temporal changes in the ratio of induced to total cells were fitted by a population dynamics model as described below. To estimate the switching rate in the absence of tryptophan, the lag (wait) time of induced cells from suppressed cells was calculated. The reverse of the mean wait time was divided by the population size (1,000 cells) to estimate the switching rate according to the population dynamics model.

### Evaluation of the population adaptation of the changing population size

To estimate the probability of the successful population adaptation, both the initial population size and the TMG concentration were varied. The initially suppressed cell populations were prepared as follows. The cells preliminarily cultured in the presence of tryptophan and various concentrations of TMG (200, 300, 400 and 500 µM) (Figure 2A, bottom panel), and were sorted into tryptophan-free media of the same TMG concentration in populations of 1 to 1,000 cells. The populations of initially induced cells were prepared by sorting the cells preliminarily cultured in the presence of 200 µM TMG with induced memory (Figure 2A, upper panel) into tryptophan-supplemented media in the numbers of 2 to 2,000 cells. Furthermore, populations of initially suppressed cells were additionally prepared by sorting the cells preliminarily cultured in the presence of 200 µM TMG but with suppressed memory (Figure 2A, bottom panel) into tryptophan-supplemented media in the numbers of 2 to 2,000 cells. Three or 12 replicates for initially induced or suppressed cells were performed, respectively. After culturing at 37°C for 1 week, the numbers of adapted and maladapted populations were counted. The criterion of adaptation was a cell concentration exceeding 10^7^ cells/mL, which was detectable by eye. The probability of population adaptation was the ratio of the number of adapted to total populations.

### Population dynamics model

We used the general model [19], which consists of two differential equations (1.1 and 1.2) that characterise the dynamics of the number of cells in the induced and suppressed states, *N_ON_* and *N_OFF_*, respectively.
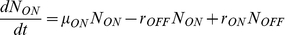
(1.1)

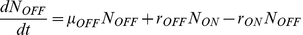
(1.2)


The parameters *r_ON_* and *r_OFF_* characterise the rates of the transition from suppressed to induced state and *vice versa*. The values *µ_ON_* and *µ_OFF_* are the instantaneous growth rates of the induced and suppressed phenotypes and depend on the nutritional environment. The ratio of the induced cells, *n_ON_*, was defined as (1.3). 
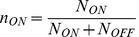
(1.3)


The differential equation regarding *n_ON_* was consequently introduced as the following equation (1.4).
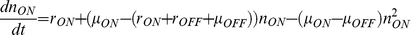
(1.4)


In the presence of intermediate concentrations of TMG (100 – 1,000 µM), the switch from induced to suppressed state was sufficiently rare to be neglected. In the presence of tryptophan, the growth rates of the suppressed and induced cells were similar (Fig. 1 d), leading to the following dynamics (1.5). These observations indicated that the timescale of the population dynamics in the presence of tryptophan is determined by the switching rate *r_ON_*. We estimated *r_ON_* in the presence of tryptophan by fitting this equation to the temporal change in the ratio of induced cells.
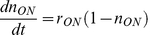
(1.5)


In the absence of tryptophan, the growth rate of the induced cells was much faster than that of the suppressed cells, resulting in the following dynamics (1.6).
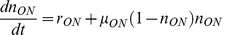
(1.6)


These observations suggest that the lag (wait) time for the first induced cell in the absence of tryptophan depends on the switching rate. The temporal change in the population dynamics is driven by the growth rate of the induced cells. Therefore, extrapolating the growth curves back enables us to estimate the wait time (when the first induced cell was born) and the switching rate *r_ON_* in the absence of tryptophan.

### Lactose utilisation network model

We used the general positive feedback model of lactose utilisation network [16], [22], [32], which consists of three differential equations of concentration of TMG, LacI and LacY, as follows (2.1, 2.2 and 2.3). The relation between TMG and LacI is determined by the following equation (2.4).
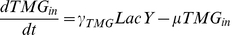
(2.1)

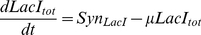
(2.2)

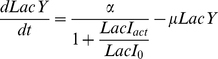
(2.3)

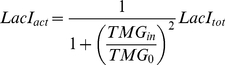
(2.4)


Here, *TMG_in_* and *LacI_tot_* are the intracellular TMG concentration and the total concentration of LacI tetramers, respectively. *LacY* is the concentration of LacY in units of green fluorescence, which is also a proxy for the relative concentration of TrpC. These components are stable in the cells and the decay is governed by the dilution due to cell growth, designated as the growth rate, *µ* (h^−1^) [22], [32]. The active fraction of LacI, denoted as *LacI_act_*, is a decreasing Hill type function of *TMG_in_* with a half-saturation concentration *TMG_0_* (M) and a Hill coefficient 2 [33]. Equation (2.1) represents the influx of TMG through the permease, LacY, with the uptake rate *γ_TMG_* (h^−1^) and the dilution *µ* (h^−1^). Equation (2.2) shows that LacI is constantly synthesised at the rate *Syn_LacI_* (Mh^−1^) and diluted by cell growth. Equation (2.3) indicates the dilution due to cell growth and the synthesis of LacY (also GFP and TrpC) as a decreasing Hill type function of *LacI_act_* with a half-saturation concentration *LacI_0_* (M) and a maximal value *α* (M h^−1^).

### Parametric phase plane

To obtain the parametric phase plane of the genetic circuit, the boundary between monostable and bistable regime was calculated [16], by combining and transforming the differential equations (2.1 – 2.3) to equation (3.1) at the steady-state.
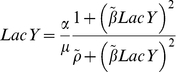
(3.1)

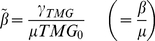
(3.2)

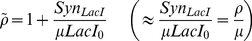
(3.3)


According to equations (3.2) and (3.3), it can be rewritten as a cubic (3.4).
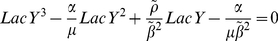
(3.4)


At the boundary between monostable and bistable regime, equation (3.4) has two identical roots and a single different root. The general cubic is described as the following equation (3.5), where *a* is the root and *θ* is the dimensionless ratio of roots.
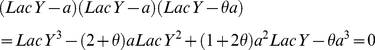
(3.5)


Comparing the coefficients, we obtained the following parametric conditions (3.6 and 3.7) to the given *θ* (>0), where the critical point is given by *θ* = 1.
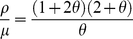
(3.6)

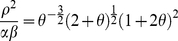
(3.7)


### Energy landscape

The temporal changes in *LacY* are described as trajectories on the energy landscape [18], [34], where *U* is energy (4.1). 
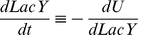
(4.1)


The energy landscape under the steady-state with regard to the other variables *TMG_in_* and *LacI* is described as the following equation (4.2). 
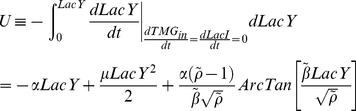
(4.2)


## Supporting Information

Figure S1 **Time lapse snapshots of switching event at the single-cell level.** Suppressed cells exposed to 300 µM TMG in the presence of tryptophan were cultured on agarose medium (1.5%) containing the same ingredients as liquid medium used in preculture. Green emission from GFP is shown in green. Induced sisters appeared, while the others remained in the suppressed state. Times are indicated in minutes.
(TIF)

Figure S2 **Population dynamics in the presence and absence of tryptophan with 400 µM TMG.** Suppressed cells exposed to 400 µM TMG were sorted and cultured in the presence or absence of tryptophan with same TMG concentration. **A**. Temporal changes in the cell populations in the presence (+Trp) and absence (−Trp) of tryptophan after cell sorting. **B**. Growth curves of cell populations in the presence (+Trp) and absence (−Trp) of tryptophan after cell sorting. The greyscale represents replicates (*n* = 2 and 10 in the presence and absence of tryptophan, respectively).
(TIF)

Figure S3 **Transformation of GFP distributions.** To estimate the ratio of the number of induced to total cells in the presence of tryptophan, dot plots (**A**) of green fluorescence (GFP FI.) over FSC for each cell population were further analysed. The corresponding transformed GFP distributions (**B**) were constructed as a spread on the red line with −4/2.6 as the slope in the upper panels. As shown in **B**, each panel represents cell populations with different TMG concentrations (0, 300, 400 and 1,000 µM TMG, respectively) in the presence of tryptophan. Red lines (**B**) represent the superimposed fit distributions of the suppressed and induced distributions (**A**). The transformed distributions applied for Figure 4 are shown, where the insets represent the ratios of the induced to total cells.
(TIF)

Figure S4 **Viability of the suppressed cells under starvation.** Suppressed cells (1000 cells/mL) grown in tryptophan-free conditions were time sampled and every 100 particles counted by cell sorter (equal to 100 µL cell culture) were plated out to the tryptophan supplied mM63 agar plates. Repeated experiments were performed. The number of the viable cells was counted as the number of the single colonies formed on the plate (colony formation unit, *cfu*) after 1 – 2 days incubation. The averaged *cfu* value of total four plates at each time point was plotted. Error bars are the standard deviations.
(TIF)

Figure S5 **Population dynamics and switching rate in response to reduced glucose.** To evaluate the native modulation of switching rate, the suppressed cells exposed to 300 µM TMG were sorted and cultured in the presence of tryptophan and 220 µM glucose. Temporal changes in cell populations after cell sorting (**A**) and the growth curve of cell population (**B**) are shown as described in Figure 3. The greyscale represents replicates (*n* = 3). Temporal changes in the ratio of the number of induced to suppressed cells during exponential growth (**C**) are given as described in Figure 4A. The solid line shows the fitting of the population dynamics model (Materials and Methods). Error bars are the standard deviations. The switching rate is indicated.
(TIF)
