# Supplementary figures and images for: Stochastic Switching Induced Adaptation in a Starved Escherichia coli Population
Source: PLoS One. 2011 Sep 13;6(9):e23953. doi: 10.1371/journal.pone.0023953 (PMC3172215; doi:10.1371/journal.pone.0023953)

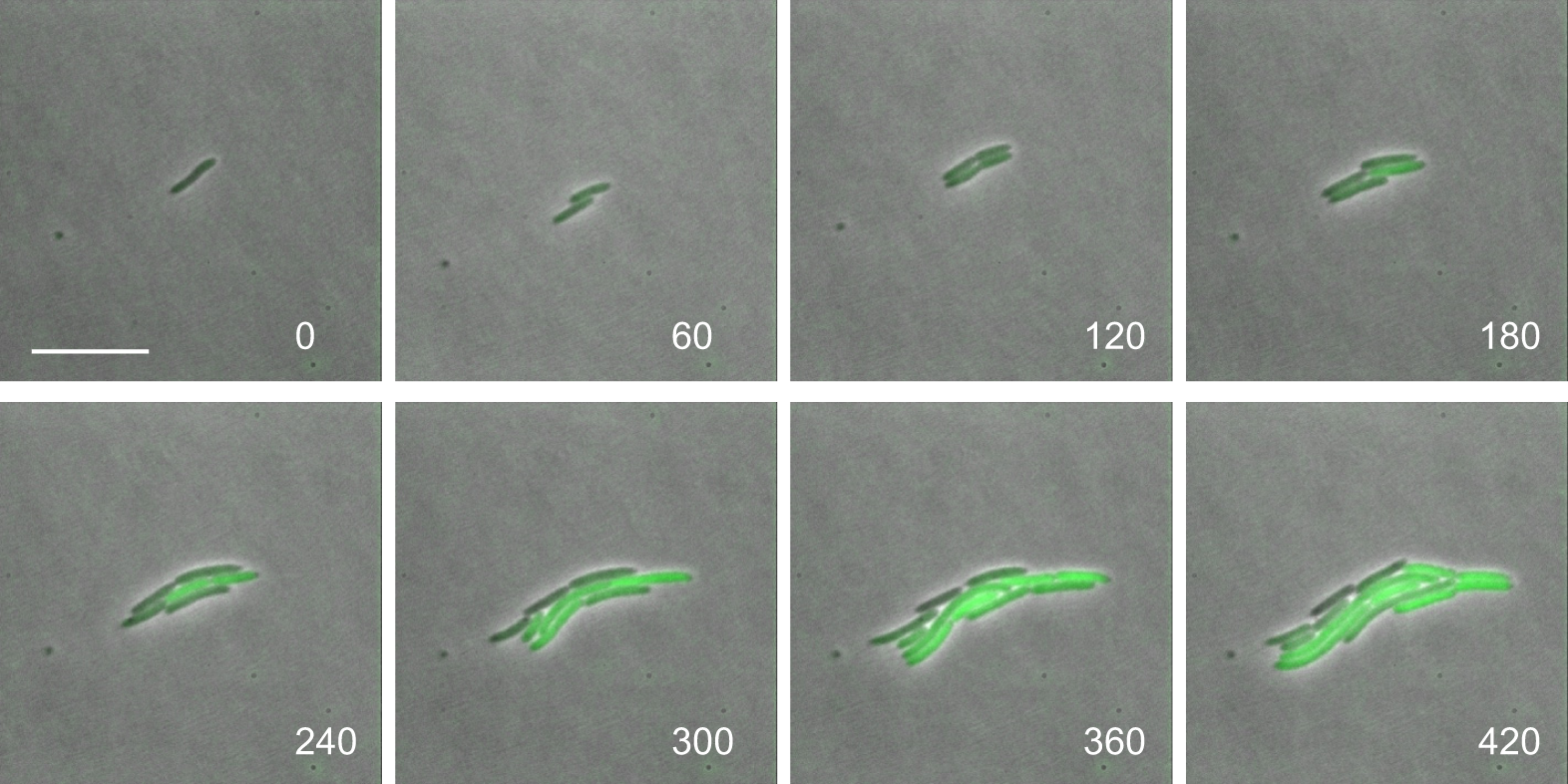

Supplement: Figure S1 — Time lapse snapshots of switching event at the single-cell level. Suppressed cells exposed to 300 µM TMG in the presence of tryptophan were cultured on agarose medium (1.5%) containing the same ingredients as liquid medium used in preculture. Green emission from GFP is shown in green. Induced sisters appeared, while the others remained in the suppressed state. Times are indicated in minutes. (TIF) [file pone.0023953.s001.tif]

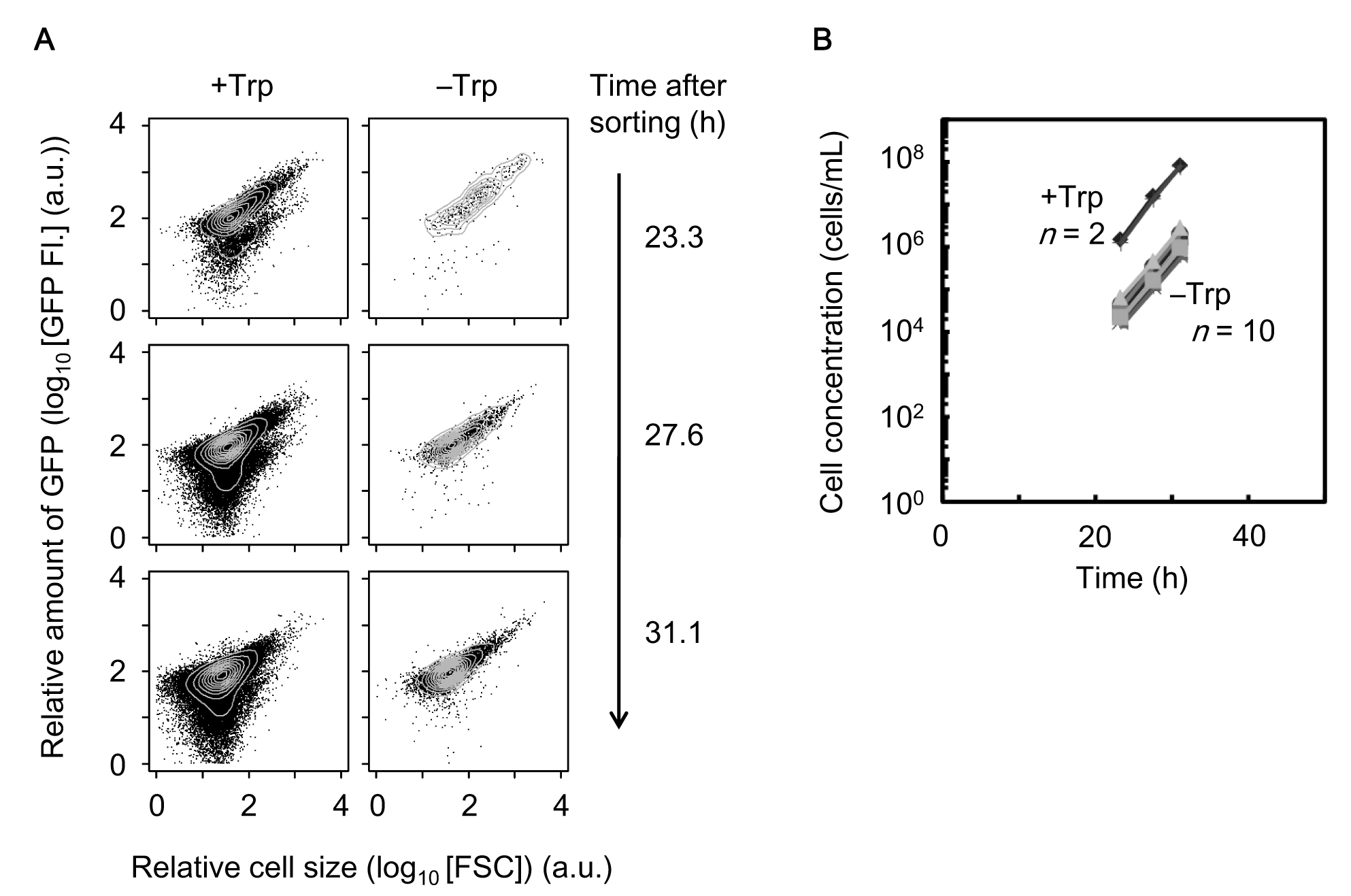

Supplement: Figure S2 — Population dynamics in the presence and absence of tryptophan with 400 µM TMG. Suppressed cells exposed to 400 µM TMG were sorted and cultured in the presence or absence of tryptophan with same TMG concentration. A. Temporal changes in the cell populations in the presence (+Trp) and absence (−Trp) of tryptophan after cell sorting. B. Growth curves of cell populations in the presence (+Trp) and absence (−Trp) of tryptophan after cell sorting. The greyscale represents replicates (n = 2 and 10 in the presence and absence of tryptophan, respectively). (TIF) [file pone.0023953.s002.tif]

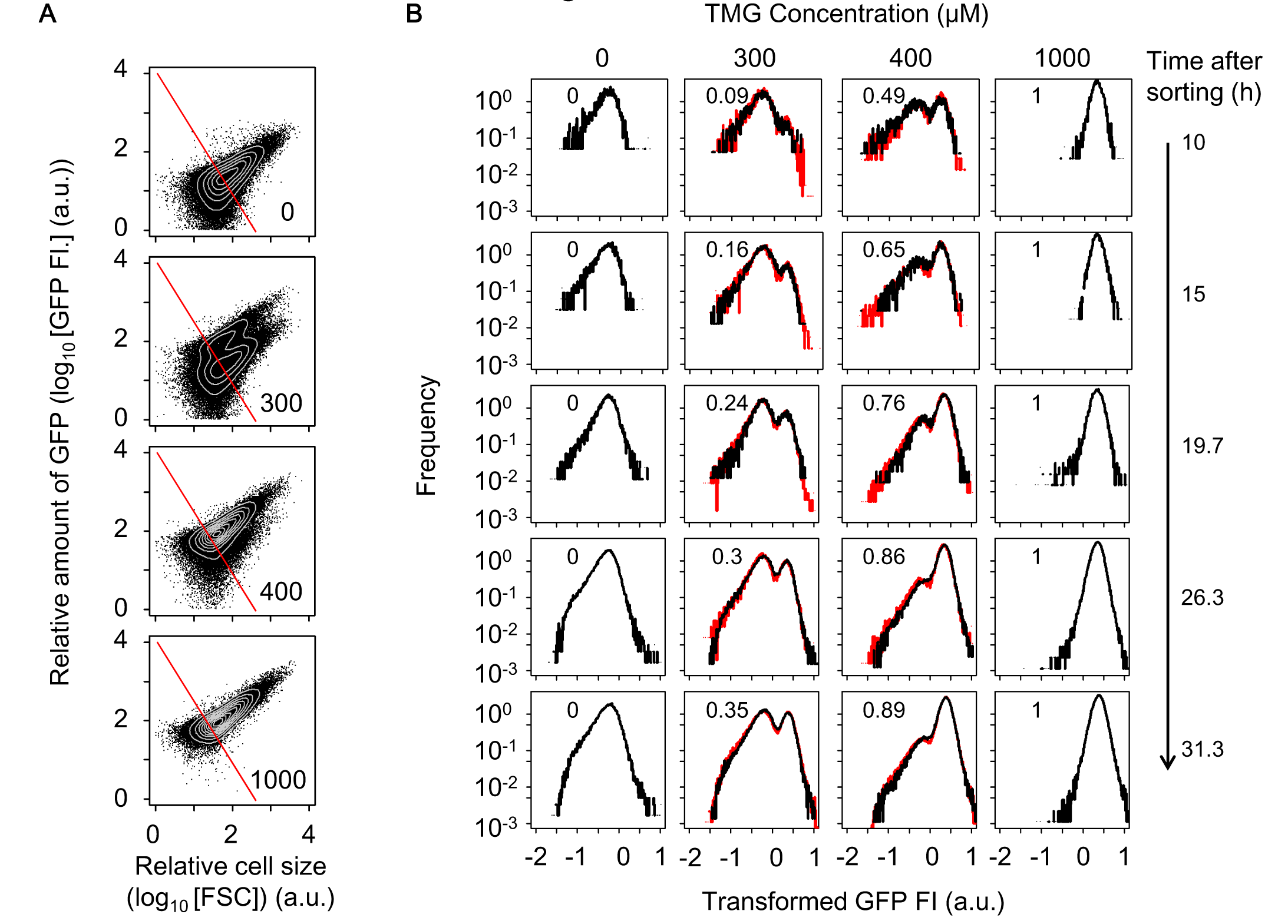

Supplement: Figure S3 — Transformation of GFP distributions. To estimate the ratio of the number of induced to total cells in the presence of tryptophan, dot plots (A) of green fluorescence (GFP FI.) over FSC for each cell population were further analysed. The corresponding transformed GFP distributions (B) were constructed as a spread on the red line with −4/2.6 as the slope in the upper panels. As shown in B, each panel represents cell populations with different TMG concentrations (0, 300, 400 and 1,000 µM TMG, respectively) in the presence of tryptophan. Red lines (B) represent the superimposed fit distributions of the suppressed and induced distributions (A). The transformed distributions applied for Figure 4 are shown, where the insets represent the ratios of the induced to total cells. (TIF) [file pone.0023953.s003.tif]

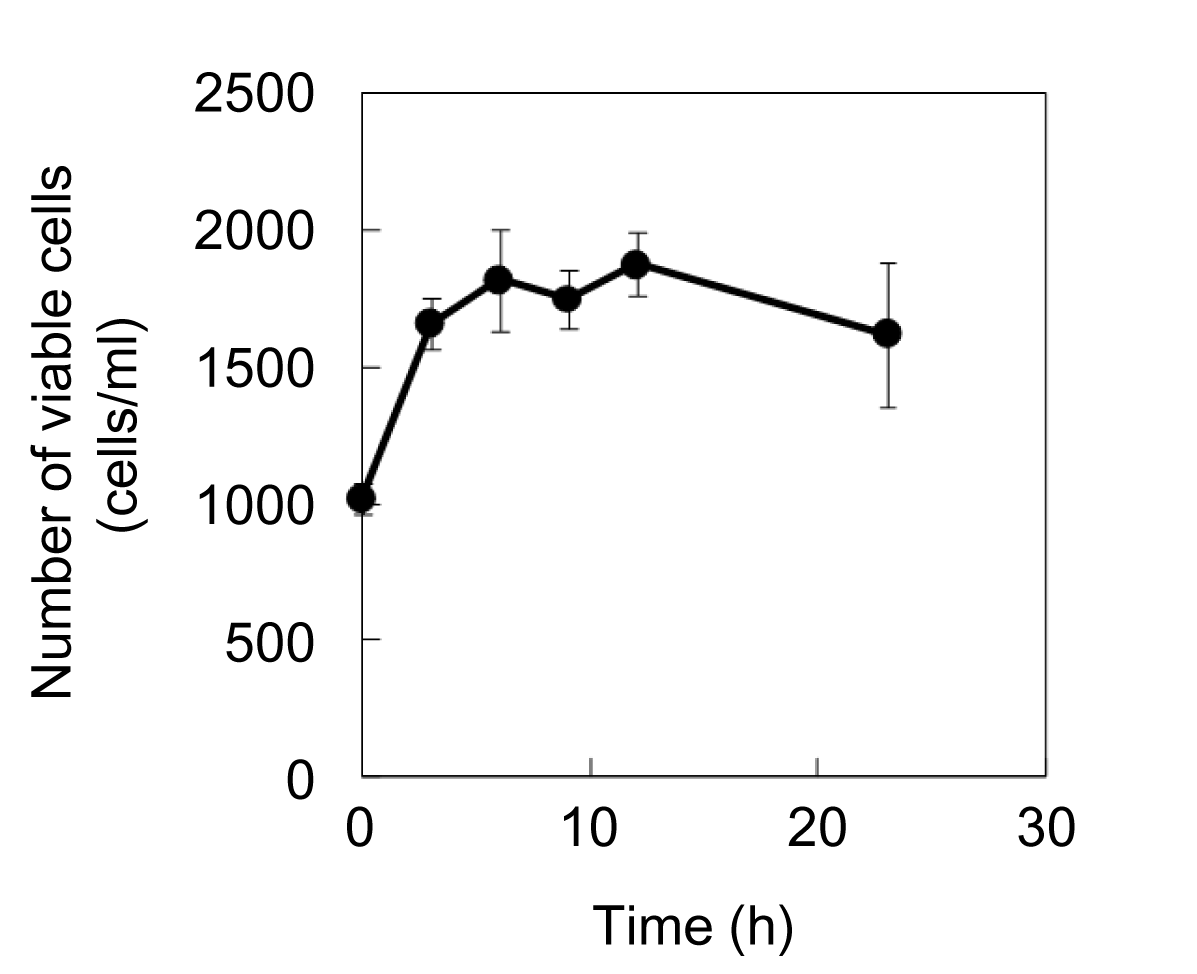

Supplement: Figure S4 — Viability of the suppressed cells under starvation. Suppressed cells (1000 cells/mL) grown in tryptophan-free conditions were time sampled and every 100 particles counted by cell sorter (equal to 100 µL cell culture) were plated out to the tryptophan supplied mM63 agar plates. Repeated experiments were performed. The number of the viable cells was counted as the number of the single colonies formed on the plate (colony formation unit, cfu) after 1 – 2 days incubation. The averaged cfu value of total four plates at each time point was plotted. Error bars are the standard deviations. (TIF) [file pone.0023953.s004.tif]

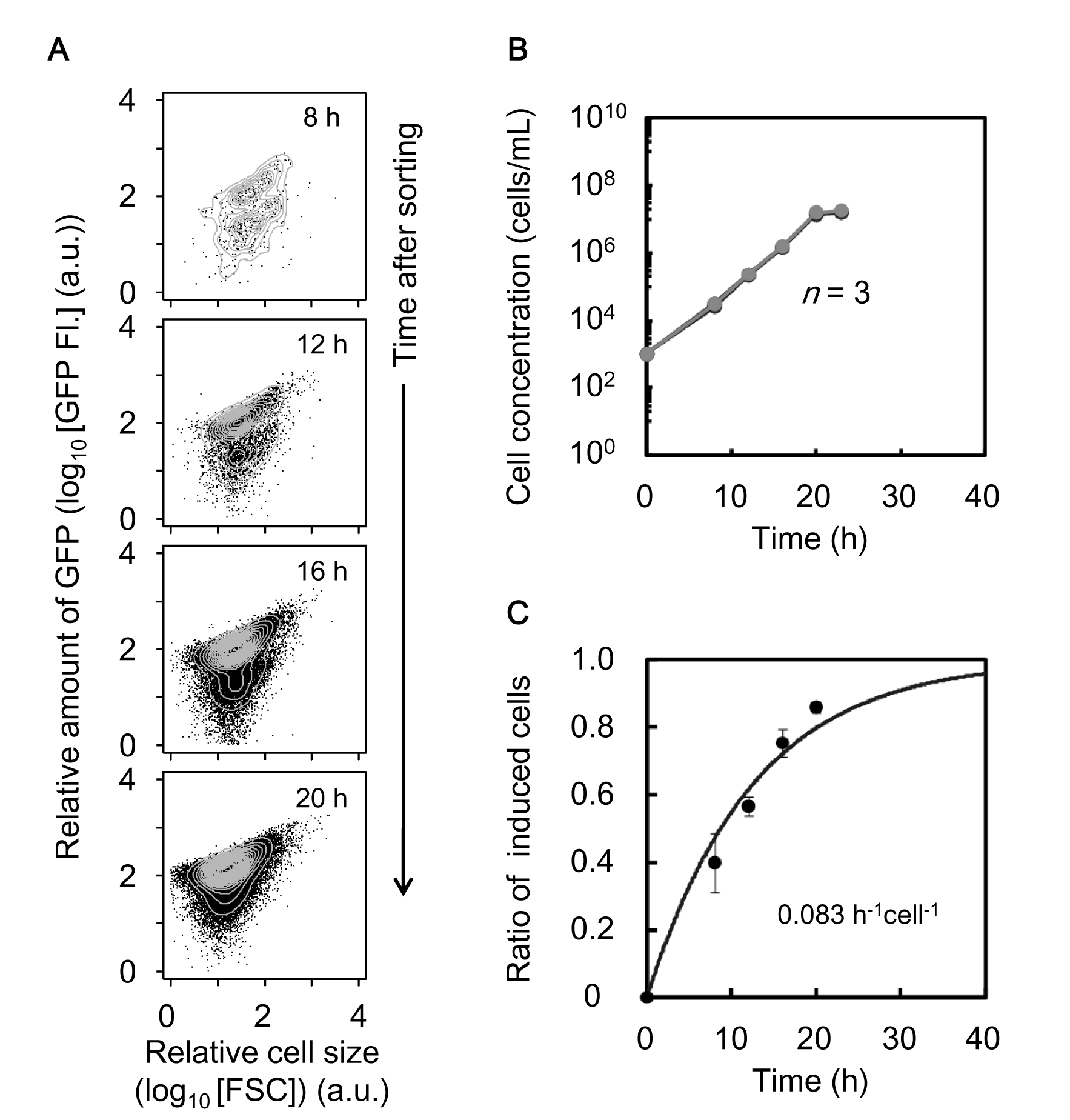

Supplement: Figure S5 — Population dynamics and switching rate in response to reduced glucose. To evaluate the native modulation of switching rate, the suppressed cells exposed to 300 µM TMG were sorted and cultured in the presence of tryptophan and 220 µM glucose. Temporal changes in cell populations after cell sorting (A) and the growth curve of cell population (B) are shown as described in Figure 3. The greyscale represents replicates (n = 3). Temporal changes in the ratio of the number of induced to suppressed cells during exponential growth (C) are given as described in Figure 4A. The solid line shows the fitting of the population dynamics model (Materials and Methods). Error bars are the standard deviations. The switching rate is indicated. (TIF) [file pone.0023953.s005.tif]
